# Romanov type problems

**DOI:** 10.1007/s11139-017-9972-8

**Published:** 2018-02-08

**Authors:** Christian Elsholtz, Florian Luca, Stefan Planitzer

**Affiliations:** 1 Institute of Analysis and Number Theory, Graz University of Technology, Kopernikusgasse 24, 8010 Graz, Austria; 2 School of Mathematics, Wits University, Private Bag X3, Wits, Johannesburg, 2050 South Africa; 3 Department of Mathematics, Faculty of Sciences, University of Ostrava, 30. dubna 22, 701 03 Ostrava 1, Czech Republic

**Keywords:** Romanov’s theorem, Smooth numbers, Diophantine equation, Sumsets, Primary 11P32, Secondary 11A07, 11A41, 11N36

## Abstract

Romanov proved that the proportion of positive integers which can be represented as a sum of a prime and a power of 2 is positive. We establish similar results for integers of the form $n=p+2^{2^k}+m!$ and $n=p+2^{2^k}+2^q$ where $m,k \in \mathbb {N}$ and *p*, *q* are primes. In the opposite direction, Erdős constructed a full arithmetic progression of odd integers none of which is the sum of a prime and a power of two. While we also exhibit in both cases full arithmetic progressions which do not contain any integers of the two forms, respectively, we prove a much better result for the proportion of integers not of these forms: (1) The proportion of positive integers not of the form $p+2^{2^k}+m!$ is larger than $\frac{3}{4}$. (2) The proportion of positive integers not of the form $p+2^{2^k}+2^q$ is at least $\frac{2}{3}$.

## Introduction

An old result of Romanov [16] states that a positive proportion of the positive integers can be written in the form $p+g^k$, where *p* is a prime and $g \ge 2$ is a positive integer. As there are about  primes $p \le x$ and  powers $g^k \le x$, this result implicitly gives some information about the number *r*(*n*) of representations of $n=p+g^k$. There are not too many integers $n \le x$ with a very large number of representations and on average *r*(*n*) is bounded. The most prominent special case of Romanov’s result is the one concerning sums of primes and powers of 2. Euler [9] observed in a letter to Goldbach that 959 can not be written as the sum of a prime and a power of two. Euler’s letter was also mentioned by de Polignac [3] and provides a counter example to a conjecture of de Polignac himself, stating that any odd positive integer is the sum of a prime and a power of 2. In 1950, Erdős [5] and van der Corput [18] independently proved that also the lower density of odd integers not of the form $p+2^k$ is positive. Here and in the following the lower density of a set $\mathcal {A} \subset \mathbb {N}$ is defined to beReplacing $\liminf$ with $\limsup$ leads to what we call upper density and if lower and upper density coincide we speak of the density of the set $\mathcal {A}$.

Concerning Romanov’s theorem one may ask how this result can be generalized. One way would be by replacing the sequence of powers of *g* with another sequence $(a_n)_{n \ge 1}$. Generalizing a result of Lee [13] who replaced the powers of *g* by the Fibonacci sequence, Ballot and Luca [1] proved an analogue of Romanov’s theorem for the case when $(a_n)_{n \ge 1}$ is a linearly recurrent sequence with certain additional properties. For certain quadratic recurrences $(a_n)_{n\ge 1}$ this was done by Dubickas [4].

We would expect that for many sets $\mathcal {A} \subset \mathbb {N}$, with $|\mathcal {A}\cap [1,x]| \ge c\log x$ for some positive constant *c*, one can write a positive proportion of integers $n \le x$ as $n=p+a$, *p* prime and $a \in \mathcal {A}$. In this paper we study sets $\mathcal {A}$ with $|\mathcal {A}\cap [1,x]| \sim c_{\mathcal {A}} \log x$ but of a quite different nature compared to previous ones. In particular, we study

$$\begin{aligned} \mathcal {A}_1&=\{2^{2^k}+m!:k,m \in \mathbb {N}_0\}, \\ \mathcal {A}_2&=\{2^{2^k}+2^q: k \in \mathbb {N}_0, q \text { prime}\}. \end{aligned}$$

Using the machinery of Romanov [16], we prove the following two theorems.

### Theorem 1

The lower density of integers of the form $p+2^{2^k}+m!$ for $k,m \in \mathbb {N}_0$ and *p* prime is positive.

### Theorem 2

The lower density of integers of the form $p+2^{2^k}+2^q$ for $k \in \mathbb {N}_0$ and *p*, *q* prime is positive.

Concerning integers not of the form $p+2^{2^k}+m!$ we consider two different questions: The first one is finding a large set, in the sense of lower density, of odd positive integers not of this form.

The second question is if there is a full arithmetic progression of odd positive integers not of the form $p+2^{2^k}+m!$. The positive answer to this question is given in Theorem 4. Note that, the density of the set constructed in the proof of Theorem 4 is considerably less than the density of the set used in the proof of Theorem 3.

### Theorem 3

The lower density of odd positive integers not of the form $p+2^{2^k}+m!$ for $k,m \in \mathbb {N}_0$ and *p* prime is at least . The lower density of all positive integers without a representation of the form $p+2^{2^k}+m!$ is therefore larger than .

### Theorem 4

There exists a full arithmetic progression of odd positive integers not of the form $p+2^{2^k}+m!$ for $k,m \in \mathbb {N}_0$ and *p* prime.

Finally, we prove analogous results for integers not of the form $p+2^{2^k}+2^q$.

### Theorem 5

There exists a subset of the odd positive integers not of the form $p+2^{2^k}+2^q$, for $k \in \mathbb {N}$ and *p*, *q* prime, with lower density . The lower density of all positive integers without a representation of the form $p+2^{2^k}+2^q$ is therefore larger than .

Furthermore, there exists a full arithmetic progression of odd positive integers not of the form $p+2^{2^k}+2^q$.

Concerning the last result, we recall that Erdős conjectured that the lower density of the set of positive odd integers not of the form $p+2^k+2^m$ is positive for $k,m \in \mathbb {N}_0$, *p* prime (see for example [10, Sect. A19]).

For the proofs of Theorems 1 and 2, we apply the method of Romanov [16]. This means that we start with the Cauchy–Schwarz inequality in the form

1 $$\begin{aligned} \left( \sum _{\begin{array}{c} n \le x \\ r_i(n)>0 \end{array}}1\right) \left( \sum _{n \le x}r_i(n)^2 \right) \ge \left( \sum _{n \le x}r_i(n)\right) ^2 \end{aligned}$$

for $i \in \{1,2\}$, where $r_1(n)$ denotes the number of representations of *n* in the form $p+2^{2^k}+m!$, and $r_2(n)$ counts the number of representations of *n* in the form $p+2^{2^k}+2^q$. Note that the first sum on the left-hand side of Eq. (1) equals the number of integers less than *x* having a representation of the required form. It thus suffices to check that

$$\begin{aligned} \sum _{n \le x}r_i(n) \gg x \text { and } \sum _{n \le x}r_i(n)^2 \ll x \end{aligned}$$

for both $i=1,~2$ in order to get positive lower density for the sets of those integers. The estimates $\sum _{n \le x}r_1(n) \gg x$ and $\sum _{n \le x}r_1(n)^2 \ll x$ are proved in Sect. 3, Lemmas 3 and 4, respectively. The analogous results for $r_2(n)$ are proved in Sect. 4, Lemmas 5 and 6, respectively. Theorems 3 and 4 are proved at the end of Sect. 3 and Theorem 5 at the end of Sect. 4.

## Notation

Let $\mathbb {N}$, as usual, denote the set of positive integers, $\mathbb {N}_0$ the set of non-negative integers and let $\mathbb {P}$ denote the set of primes. The variables *p* and *q* will always denote prime numbers. For any prime $p \in \mathbb {P}$ and any positive integer $n \in \mathbb {N}$, let $\nu _p(n)$ denote the *p*-adic valuation of *n*, i.e. $\nu _p(n)=k$ where $p^k$ is the highest power of *p* dividing *n*. For an integer *n*, *P*(*n*) denotes its largest prime factor. For any set $S \subset \mathbb {N}$ let $S(x) = |S \cap [1,x]|$ denote the counting function of *S*. As usual $\varphi$ denotes Euler’s totient function and $\mu$ the Möbius function. Furthermore, for an odd positive integer *n* we denote by *t*(*n*) the order of $2 \bmod n$. We use the symbols $\ll$, $\gg$, $\mathcal {O}$ and *o* within the context of the well-known Vinogradov and Landau notation.

## Integers of the form $p+2^{2^k}+m!$

Before proving Lemmas 3 and 4, we establish and collect several results needed in due course. The following is a classical result due to Legendre (see for example Theorems 2.6.1 and 2.6.4 in [14]).

### Lemma A

For any prime $p \in \mathbb {P}$ and any positive integer $n \in \mathbb {N}$, we have that

$$\begin{aligned} \nu _p(n!) = \sum _{k=1}^{\infty }\left\lfloor \frac{n}{p^k}\right\rfloor . \end{aligned}$$

Furthermore, if $\sigma _p(n)$ denotes the sum of base *p* digits of *n*, then

$$\begin{aligned} \nu _p(n!) = \frac{n-\sigma _p(n)}{p-1}. \end{aligned}$$

### Theorem 6

The equation $2^{x_1}+y_1!=2^{x_2}+y_2!$ has only four non-negative integer solutions $(x_1,y_1,x_2,y_2)$ with $x_1>x_2$ where either $x_2 \le 52$ or $y_2 \le 8$. These solutions are

$$\begin{aligned} (x_1,y_1,x_2,y_2) \in \{(1,0,0,2),(1,1,0,2),(3,2,2,3),(7,4,5,5)\}. \end{aligned}$$

### Proof

Suppose that $x_2 \le 52$ and note that $y_1=0$ either implies that $y_2\in \{0,1\}$ if $x_2>0$, which leads to a solution where $x_1=x_2$, which is excluded, or implies that $x_2=0$, whence $x_1=1$ and $y_2=2$. Hence, the only solution where $y_1=0$ is $(x_1,y_1,x_2,y_2)=(1,0,0,2)$. From now on, we may suppose that $y_1 \ge 1$. In this case, from Lemma A, we get that $\nu _2(y_1!) \ge \frac{y_1}{2}-1$. This yields $\frac{y_1}{2}-1 \le x_2$ and thus $y_1 \le 106$. Since

$$\begin{aligned} 2^{x_2}-y_1!=2^{x_1}-y_2!, \end{aligned}$$

we have $\nu _2(2^{x_2}-y_1!)=\nu _2(2^{x_1}-y_2!)$. Certainly $|2^{x_2}-y_1!| \le 2^{52}+106!$ which implies that $\nu _2(2^{x_2}-y_1!) \le \frac{\log (2^{52}+106!)}{\log 2}<816$. If $x_1\ge 816$ and $y_2 \ge 822$, then $\nu _2(2^{x_1}-y_2!)\ge 816$, a contradiction. The cases where either $x_1 \le 815$ or $y_2 \le 821$ can be checked by a computer search which leads to the solutions

$$\begin{aligned} (x_1,y_1,x_2,y_2) \in \{(1,0,0,2),(1,1,0,2),(3,2,2,3),(7,4,5,5)\}. \end{aligned}$$

Now suppose that $y_2 \le 8$ and consider

$$\begin{aligned} 0<2^{x_1}-2^{x_2}=y_2!-y_1!, \end{aligned}$$

which implies that $y_1 \le y_2 \le 8$. In particular, $|y_2!-y_1!|\le 2\cdot 8!$ and thus

$$\begin{aligned} \nu _2(y_2!-y_1!) \le \frac{\log (2\cdot 8!)}{\log 2} < 17. \end{aligned}$$

Since $\nu _2(2^{x_1}-2^{x_2})=x_2$, we have that $x_2 < 17$ which is included in the case $x_2 \le 52$ treated above. $\square$

### Theorem 7

If we exclude solutions arising from interchanging $(x_1,y_1)$ and $(x_2,y_2)$, the equation $2^{x_1}+y_1!=2^{x_2}+y_2!$ has only four non-negative integer solutions $(x_1,y_1,x_2,y_2)$ with $(x_1,y_1) \ne (x_2,y_2)$ and $(y_1,y_2) \not \in \{(1,0),(0,1)\}$ if $x_1=x_2$. These are the solutions presented in Theorem 6.

### Proof

We compare the 2-adic and 3-adic valuation of both sides of equivalent forms of the equation $2^{x_1}+y_1!=2^{x_2}+y_2!$ to get information about the size of the parameters $x_1,x_2,y_1$ and $y_2$.

If $x_1=x_2$ we have that $y_1!=y_2!$ and hence either $y_1=y_2$ or $(y_1,y_2) \in \{(1,0),(0,1)\}$ which leads to the excluded trivial solutions. Therefore, w.l.o.g., we may suppose that $x_1>x_2$ and write

2 $$\begin{aligned} 2^{x_2}(2^{x_1-x_2}-1)=y_1!((y_1+1) \cdots y_2-1). \end{aligned}$$

Next we compute the 2-adic valuation of both sides of the last equality. For the left-hand side we simply have $\nu _2(2^{x_2}(2^{x_1-x_2}-1))=x_2$ while for the right-hand side we use that the factor $((y_1+1)\cdots y_2-1)$ is odd as soon as $y_2 \ge y_1+2$ which yields

$$\begin{aligned} \nu _2(y_1!((y_1+1)\cdots y_2-1)) ={\left\{ \begin{array}{ll} \nu _2(y_1!), &{}\text { if } y_2 \ge y_1+2, \\ \nu _2(y_1!)+\nu _2(y_1), &{}\text { if } y_2=y_1+1. \end{array}\right. } \end{aligned}$$

From this, Lemma A and the fact that $1 \le \sigma _2(y_1) \le \frac{\log y_1}{\log 2}+1$ (note that as in the proof of Theorem 6, $y_1 \in \{0,1\}$ leads to a single non-trivial solution listed there), we get the following two inequalities:

3 $$\begin{aligned} x_2=\nu _2(2^{x_2}(2^{x_1-x_2}-1))&=\nu _2(y_1!((y_1+1)\cdots y_2-1)) \le \nu _2(y_1!)+\nu _2(y_1) \nonumber \\&<y_1+\frac{\log y_1}{\log 2}, \end{aligned}$$

4 $$\begin{aligned} x_2=\nu _2(2^{x_2}(2^{x_1-x_2}-1))&=\nu _2(y_1!((y_1+1)\cdots y_2-1)) \ge \nu _2(y_1!) \nonumber \\&\ge y_1-\left( \frac{\log y_1}{\log 2}+1\right) . \end{aligned}$$

By Theorem 6, we may suppose that $x_2 \ge 5$ without loosing solutions. In this case, the last inequality implies $y_1 \le 2x_2$.

Next, we look at

$$\begin{aligned} 2^{x_1}=2^{x_2}+y_2!-y_1!. \end{aligned}$$

Since $2^{x_2} \le 2^{x_1-1}=\frac{2^{x_1}}{2}$, we have that $y_2!>\frac{2^{x_1}}{2}$, whence we get

$$\begin{aligned} y_2^{y_2} \ge y_2!>\frac{2^{x_1}}{2}, \end{aligned}$$

and thus

$$\begin{aligned} y_2\log y_2> (x_1-1)\log 2 \text { and } y_2>\frac{(x_1-1)\log 2}{\log y_2}. \end{aligned}$$

To get the last inequality we used that by Theorem 6 we may suppose that $y_2 \ge 9$ whence $\log y_2 >0$. Now $x_2 \ge 5$ implies that $x_1 \ge 6$. If we would have that $y_2 \le x_1$ the last inequality would imply that

5 $$\begin{aligned} y_2> \frac{\log 2}{2}\left( \frac{x_1}{\log y_2}\right) >\frac{1}{4}\left( \frac{x_1}{\log x_1}\right) . \end{aligned}$$

In order to prove (5), it therefore suffices to prove that $y_2 \le x_1$ for $x_1 \ge 6$. In order to do so, we consider the equation

$$\begin{aligned} 2^{x_1}=y_1!((y_1+1)\cdots y_2-1)+2^{x_2} \end{aligned}$$

from which we readily deduce that $y_1! <2^{x_1}$. This together with $2^{x_1}=y_2!-y_1!+2^{x_2}$ implies that

$$\begin{aligned} y_2!<2\cdot 2^{x_1}. \end{aligned}$$

This implies that $y_2 \le x_1$, since otherwise $(x_1+1)! \le 2^{x_1+1}$ which is true for $x_1 \le 2$ only. By Theorem 6 again, we may suppose that $y_2 \ge 9$. In this case, applying Lemma A, we obtain

6 $$\begin{aligned} \nu _3(y_2!) \ge \left\lfloor \frac{y_2}{3}\right\rfloor +\left\lfloor \frac{y_2}{9} \right\rfloor \ge \frac{y_2}{3} > \frac{1}{12}\left( \frac{x_1}{\log x_1}\right) , \end{aligned}$$

where the last inequality follows by (5). Now we compute the 3-adic valuation of both sides of Eq. (2). By inequality (3) and Lemma A for the right-hand side, we get

$$\begin{aligned} k=\nu _3(y_1!((y_1+1)\cdots y_2-1))&\ge \nu _3(y_1!)=\frac{y_1-\sigma _3(y_1)}{2}\ge \frac{y_1}{2}-\frac{\log y_1}{\log 3}-1 \\&\ge \frac{x_2}{2}-\log (y_1)\left( \frac{1}{2\log 2}+\frac{1}{\log 3}\right) -1. \end{aligned}$$

Since for the left-hand side of (2) we have $3^k|2^{x_1-x_2}-1$, we deduce that $\varphi (3^k)=2\cdot 3^{k-1}|x_1-x_2$. Here we used that 2 is a primitive root modulo any power of 3. This is a direct consequence of Jacobi’s observation [12, p. XXXV] that a primitive root modulo $p^2$ is also a primitive root modulo any higher power of *p*. Using the above bound for *k* and the fact that $y_1 \le 2x_2$, we get7Next we find an upper bound for $x_1$ in terms of $x_2$. Consider the equation

$$\begin{aligned} 2^{x_1}-y_2!=2^{x_2}-y_1!. \end{aligned}$$

Equation (5) yields that $y_2> \frac{1}{4}\frac{x_1}{\log x_1}>\frac{1}{4} \sqrt{x_1}.$ Thus, by Lemma A, $\nu _2(y_2!)>\frac{\sqrt{x_1}}{8}-1$ and hence $\nu _2(2^{x_1}-y_2!)\ge \frac{\sqrt{x_1}}{8}-1$.

On the other hand, $|2^{x_2}-y_1!|\le 2^{x_2}+y_1! \le 2^{x_2}+(2x_2)^{2x_2}\le 2\cdot (2x_2)^{2x_2}$. Now $\nu _2(2^{x_2}-y_1!)$ is certainly bounded from above by the highest power of 2 less than $2\cdot (2x_2)^{2x_2}$:

$$\begin{aligned} 2^a \le 2\cdot (2x_2)^{2x_2} \Leftrightarrow a \le \frac{2x_2\log (2x_2)}{\log 2}+1. \end{aligned}$$

We therefore have that $\nu _2(2^{x_2}-y_1!) \le 4x_2\log (2x_2)+1$ and putting everything together, we get

$$\begin{aligned} \frac{\sqrt{x_1}}{8}-1 \le \nu _2(2^{x_1}-y_2!)=\nu _2(2^{x_2}-y_1!)\le 4x_2\log (2x_2)+1, \end{aligned}$$

which implies that $x_1 \le (32x_2\log (2x_2)+16)^2$. Combining this with (7), we finally arrive atThis inequality is valid only for $x_2 \le 52$ and the solutions satisfying this restriction are given in Theorem 6. $\square$

### Lemma 1

Let $m_1,m_2,m_3,m_4 \in \mathbb {N}$ such that $m_1>m_2$, $m_3>m_4$ and

8 $$\begin{aligned} m_1!-m_2!=m_3!-m_4!. \end{aligned}$$

Then $(m_1,m_2)=(m_3,m_4)$ or $m_1=m_3$ and $(m_2,m_4) \in \{(0,1),(1,0)\}$.

### Proof

We start with the case where either $m_1=m_2+1$ or $m_3=m_4+1$ and w.l.o.g suppose that $m_1=m_2+1$. If furthermore $m_2 \le m_4$, we get from Eq. (8)

$$\begin{aligned} m_2!\, m_2=m_4!((m_4+1)\cdots m_3-1) \ge m_2!m_4, \end{aligned}$$

which leads to $m_2 \ge m_4$ and thus $m_2=m_4$ which implies $m_1=m_3$. On the other hand, if $m_1=m_2+1$ and $m_2>m_4$ Eq. (8) implies that

9 $$\begin{aligned} m_2(m_4+1)\cdots m_2=(m_4+1) \cdots m_3-1, \end{aligned}$$

and therefore $m_4+1|1$ if $m_3>m_4+1$ and $m_4+1|m_4$ otherwise, whence $m_4=0$ in both cases. Now $m_3=1$ implies that $(m_1,m_2)=(1,0)$ and we are done. Otherwise, if $m_3 \ne 1$, then the right-hand side of (9) is odd. In order for the left-hand side to be odd we need $m_2=1$, which implies that $m_1=m_3$.

It remains to consider the case where $m_1\ge m_2+2$ and $m_3 \ge m_4+2$ and w.l.o.g. we suppose that $m_2 > m_4$. We look at Eq.  (8) in the form

10 $$\begin{aligned} m_2!((m_2+1)\cdots m_1-1)=m_4!((m_4+1)\cdots m_3-1). \end{aligned}$$

By assumption, we have that $\nu _2(m_2!)=\nu _2(m_4!)$ which implies that $m_4$ is even and $m_2=m_4+1$. We hence may rewrite Eq.  (10) to get

$$\begin{aligned} (m_4+1)\cdots m_1 -m_4=(m_4+1)\cdots m_3. \end{aligned}$$

It follows that $m_4+1|m_4$ which implies that $m_4=0$. This leads to $m_2=1$ and $m_1=m_3$. $\square$

### Lemma 2

For odd positive *n*, let *t*(*n*) be the order of $2 \bmod n$ and $t(n)=2^{a(n)}b(n)$ such that *b*(*n*) is odd. Then the series

$$\begin{aligned} \sum _{\begin{array}{c} 2 \not \mid n \\ \mu ^2(n)=1 \end{array}} \frac{1}{nt(b(n))} \end{aligned}$$

converges.

### Proof

Recall that *P*(*n*) denotes the largest prime factor of *n* and observe that if *u*|*v* then *t*(*u*)|*t*(*v*), thus *b*(*u*)|*b*(*v*) and further *t*(*b*(*u*))|*t*(*b*(*v*)). From this and Mertens’ formula in the weak form

$$\begin{aligned} \prod _{p \le x}\left( 1+\frac{1}{p}\right) \ll \log x, \end{aligned}$$

we get

11 $$\begin{aligned} \sum _{\begin{array}{c} 2 \not \mid n \\ \mu ^2(n)=1 \end{array} }\frac{1}{nt(b(n))}&\le \sum _{\begin{array}{c} p \ge 3 \\ p \in \mathbb {P} \end{array}}\frac{1}{pt(b(p))}\sum _{\begin{array}{c} 2 \not \mid m \\ \mu (m)^2=1 \\ P(m)<p \end{array}}\frac{1}{m}=\sum _{\begin{array}{c} p \ge 3 \\ p \in \mathbb {P} \end{array}}\frac{1}{pt(b(p))}\prod _{\begin{array}{c} q<p \\ q \in \mathbb {P} \end{array}}\left( 1+\frac{1}{q}\right) \nonumber \\&\ll \sum _{\begin{array}{c} p \ge 3 \\ p \in \mathbb {P} \end{array}}\frac{\log p}{pt(b(p))}. \end{aligned}$$

We split the primes into two subsets $\mathcal {P}$ and $\mathcal {Q}$ and consider the contribution of these sets separately. We set $\mathcal {P}=\mathcal {P}_1 \cup \mathcal {P}_2 \cup \mathcal {P}_3 \cup \mathcal {P}_4$ wherefor some fixed $p_0$ to be chosen later. The set $\mathcal {Q}$ is then defined to be $\mathbb {P}\backslash (\mathcal {P}\cup \{2\})$. We start by showing that

12 $$\begin{aligned} \mathcal {P}(x) \ll \frac{x}{(\log x)^3}. \end{aligned}$$

For $\mathcal {P}_1$, applying an idea of Erdős and Murty [6], we use that $p|2^k-1$ where $k=t(p)$, whence we have thatFrom this, we getwhich shows that13To deal with the contribution of the set $\mathcal {P}_2$, we set

$$\begin{aligned} {\varPsi }(x,y):=|\{n \le x: P(n) \le y\}|. \end{aligned}$$

By known results on smooth numbers (in particular, a result of Canfield, Erdős and Pomerance from [2, Corollary p.15]), we have for $y>(\log x)^2$,

14 $$\begin{aligned} {\varPsi }(x,y)=\frac{x}{\exp ((1+o(1))u\log u)},\quad \text { where }\quad u=\frac{\log x}{\log y}, \end{aligned}$$

as both *y* and *u* tend to infinity. For $p \in \mathcal {P}_2$ we may suppose that  since there are at most  primes in $\mathcal {P}_2$ less than $\sqrt{x}$. If , then  for sufficiently large *x*, and hence for  in ${\mathcal P}_2$, we havePut . Thus, $p-1$ is a number which is at most *x*, having a divisor , whose largest prime factor is at most *y*. It follows that $p-1\le x$ is a multiple of some number  with $P(d)\le y$. For a fixed *d*, the number of such *p* is at most . Summing over *d*, we get thatPutting , we get that  for all , and

15 $$\begin{aligned} (1+o(1))u_0\log u_0=\left( \frac{1}{12}+o(1)\right) \log \log x \log \log \log x>4\log \log x \end{aligned}$$

for large *x*. Using (14) and (15), we thus get that

$$\begin{aligned} {\mathcal P}_2(x)\ll \frac{x+x\log x}{\exp ((1+o(1))u_0\log u_0)}\ll \frac{x}{(\log x)^3}. \end{aligned}$$

Next we consider the contribution of $\mathcal {P}_3$. This set contains primes *p* such that $p-1$ is divisible by some prime  but $q \in \mathcal {P}_1$. We may assume again that , then , where as before . Fixing *q*, the number of primes $p \le x$ such that $p-1$ is a multiple of *q* is at most . Summing up over $q \in \mathcal {P}_1$ and using (13), we get thatFinally, choose $p_0$ such that for $p>p_0$ we have that  and get

$$\begin{aligned} \mathcal {P}_3(x) \ll 1 \ll \frac{x}{(\log x)^3}. \end{aligned}$$

We are now ready to prove that the sum on the right-hand side of (11) converges. For the contribution of primes $p \in \mathcal {P}$, we use the Abel summation formula as well as (12) and get

$$\begin{aligned} \sum _{\begin{array}{c} p \le x \\ p \in \mathcal {P} \end{array}}\frac{\log p}{pt(b(p))}&\le \sum _{\begin{array}{c} p \le x \\ p \in \mathcal {P} \end{array}} \frac{\log p}{p} =\int _3^x\frac{\log t}{t} \mathrm {d}\mathcal {P}(t) \\&= \frac{\mathcal {P}(t)\log t}{t}\Big |_{t=3}^x-\int _3^x\frac{1-\log t}{t^2}\mathcal {P}(t)\mathrm {d}t \\&\ll 1+ \int _3^x\frac{\log t}{t^2}\frac{t}{(\log t)^3} \mathrm {d}t =1+\int _3^x\frac{\mathrm {d}t}{t(\log t)^2} \ll 1. \end{aligned}$$

By the definition of $\mathcal {Q}$ for $p \in \mathcal {Q}$ we have that  which implies that *q*|*b*(*p*) for large *p*. Furthermore, $q \not \in \mathcal {P}_1$ so . By the choice of the constant $p_0$ in the definition of $\mathcal {P}_4$ this implies that $t(b(p))\ge t(q) >(\log p)^3$. Finally, this implies that

$$\begin{aligned} \sum _{p \in Q}\frac{\log p}{pt(b(p))} \le \sum _{n \in \mathbb {N}}\frac{1}{n(\log n)^2} \ll 1, \end{aligned}$$

which finishes the proof of the lemma. $\square$

### Lemma 3

The following estimate holds:

$$\begin{aligned} \sum _{n \le x}r_1(n) \gg x. \end{aligned}$$

### Proof

We certainly have thatBy the Prime Number Theorem16and  implies that  and hence17We use that $m! \le m^m$ and that  for  and sufficiently large *x*. This implies that18The bounds in (16), (17) and (18) show that

$$\begin{aligned} \sum _{n \le x}r_1(n) \gg x. \end{aligned}$$

$$\square $$

### Lemma 4

The following estimate holds:

$$\begin{aligned} \sum _{n \le x}r_1(n)^2 \ll x. \end{aligned}$$

### Proof

We begin with the observation that the sum counts exactly the number of solutions of the equation

$$\begin{aligned} p_1+2^{2^{k_1}}+m_1!=p_2+2^{2^{k_2}}+m_2! \end{aligned}$$

in $p_1,p_2,k_1,k_2,m_1$ and $m_2$ where $p_1+2^{2^{k_1}}+m_1! \le x$. For fixed $k_1,k_2,m_1$ and $m_2$ this amounts to counting pairs of primes $(p_1,p_2)$ such that $p_2=p_1+h$, where

$$\begin{aligned} h:=2^{2^{k_1}}+m_1!-2^{2^{k_2}}-m_2!. \end{aligned}$$

If $h=0$, then we apply Theorem 7 to get that either $(k_1,m_1)=(k_2,m_2)$ or $k_1=k_2$ and $(m_1,m_2) \in \{(1,0),(0,1)\}$^1^. The number of choices of the form $(p_1,p_2,k_1,k_2,m_1,m_2)$ in this case is

$$\begin{aligned} \mathcal {O}\left( \frac{x}{\log x}\left( \log \log x \frac{\log x}{\log \log x}+\log \log x \right) \right) =\mathcal {O}(x). \end{aligned}$$

If *h* is odd, then one of the primes $p_1$ and $p_2$ equals 2 and any choice of $(k_1,k_2,m_1,m_2)$ fixes the other prime. There are

$$\begin{aligned} \mathcal {O}\left( (\log \log x)^2 \left( \frac{\log x}{\log \log x}\right) ^2\right) =o(x) \end{aligned}$$

choices for $(p_1,p_2,k_1,k_2,m_1,m_2)$ in this case. To deal with the remaining even $h \ne 0$, we use a classical sieve bound (cf. for example [15, Theorem 7.3]). In this case, the number of pairs $(p_1,p_2)$ of primes such that $p_2=p_1+h$ is

$$\begin{aligned} \mathcal {O}\left( \frac{x}{(\log x)^2}\prod _{p|h}\left( 1+\frac{1}{p}\right) \right) . \end{aligned}$$

Summing over all choices $(k_1,k_2,m_1,m_2)$ such that $h \ne 0$ is even (this range of summation is indicated by the dash in the superscript of the sum below), we hence need to show that19Observing that the prime $p=2$ contributes just a constant factor, this amounts to showing thatwhich we do in what follows. We now rewrite the left-hand side of the last inequality above asTherefore we need to study, for a given odd square-free *d*, the cardinality of the set

$$\begin{aligned} S_d:=\{(k_1,k_2,m_1,m_2):d|h, h\ne 0, 2\not \mid h\}. \end{aligned}$$

We start with the subset $S_{1,d} \subset S_d$ where

20 $$\begin{aligned} S_{1,d}:=\{(k_1,k_2,m_1,m_2)\in S_d: m_1=m_2~{\text {or}}~\{m_1,m_2\}=\{0,1\}\}. \end{aligned}$$

We thus first deal withBy (20), $(m_1,m_2)$ is chosen in at most  ways. As for $(k_1,k_2)$, we have $2^{2^{k_1}}\equiv 2^{2^{k_2}}\pmod d$. Since *d* is odd this implies that $2^{2^{k_1}-2^{k_2}}\equiv 1\pmod d$. Recall that *t*(*d*) is the order of 2 modulo *d*. The above congruence makes $2^{k_1}\equiv 2^{k_2}\pmod {t(d)}$. As above we write $t(d)=2^{a(d)} b(d)$, where *b*(*d*) is odd and *a*(*d*) is some non-negative integer. This implies that $2^{k_1-k_2}\equiv 1\pmod {b(d)}$. The above cancellation again is justified since *b*(*d*) is odd. Hence, for $k_2$ fixed, $k_1$ is in a fixed arithmetic progression modulo *t*(*b*(*d*)). The number of such $k_1$ with $2^{2^{k_1}}\le x$ is of order (up to a constant) at most

$$\begin{aligned} \left\lfloor \frac{\log \log x}{t(b(d))}\right\rfloor +1. \end{aligned}$$

Since $k_2$ is chosen in $\mathcal {O}(\log \log x)$ ways, we havewhere we used Lemma 2 and the fact that

$$\begin{aligned} \sum _{\begin{array}{c} d \le x \\ d \text { odd} \\ \mu (d)^2=1 \end{array}}\frac{1}{d} \ll \log x. \end{aligned}$$

From now on, we deal with $S_d\backslash S_{1,d}$. Any quadruple $(k_1,k_2,m_1,m_2)$ in the above set gives $m_1!-m_2!\ne 0$ and we assume that $m_1>m_2$. We partition the numbers *d* in the range of summation into two different sets *A* and *B*. We set

$$\begin{aligned}&A:= \\&\quad \left\{ d \in \mathbb {N} :\begin{gathered} 2 \not \mid d, \mu (d)^2=1,\forall \{(k_1,k_2,m_1,m_2),(k_3,k_4,m_3,m_4)\} \in (S_d\backslash S_{1,d})^2: \\ 2^{2^{k_1}}+m_1!-2^{2^{k_2}}-m_2!=2^{2^{k_3}}+m_3!-2^{2^{k_4}}-m_4!=h \end{gathered} \right\} \!, \\&B:=\\&\quad \left\{ d \in \mathbb {N} :\begin{gathered} 2\not \mid d, \mu (d)^2=1,\exists \{(k_1,k_2,m_1,m_2),(k_3,k_4,m_3,m_4)\} \in (S_d\backslash S_{1,d})^2: \\ 2^{2^{k_1}}+m_1!-2^{2^{k_2}}-m_2!\ne 2^{2^{k_3}}+m_3!-2^{2^{k_4}}-m_4! \end{gathered} \right\} \!. \end{aligned}$$

In the set *A* we thus collect all *d* for which all solutions in $S_d\backslash S_{1,d}$ give the same *h* and the set *B* contains all other *d*. For $d \in A$ we fix $k_1$ and $k_2$ for solutions in $S_d\backslash S_{1,d}$ and get

$$\begin{aligned} m_1!-m_2!=h-2^{2^{k_1}}+2^{2^{k_2}}. \end{aligned}$$

The existence of some other element $(k_1,k_2,m_3,m_4) \in S_d\backslash S_{1,d}$ with $m_3>m_4$ would imply that $m_1!-m_2!=m_3!-m_4!$ which by Lemma 1 leads to $(m_1,m_2)=(m_3,m_4)$. Hence, for $d\in A$ and for $(k_1,k_2,m_1,m_2) \in S_d\backslash S_{1,d}$ with $m_1>m_2$, the last two coordinates are uniquely determined by the first two whence for $d \in A$ we have

$$\begin{aligned} | (S_d\backslash S_{1,d})|\ll (\log \log x)^2. \end{aligned}$$

We thus get that

$$\begin{aligned} \sum _{d\in A} \frac{|(S_d\backslash S_{1,d})|}{d} \ll (\log \log x)^2 \sum _{d\le x} \frac{1}{d}\ll (\log x)(\log \log x)^2=o((\log x)^2). \end{aligned}$$

Finally, we deal with the contribution of $d \in B$. By definition, we may find two quadruples $(k_1,k_2,m_1,m_2)$ with $m_1>m_2$ and $(k_3,k_4,m_3,m_4)$ with $m_3>m_4$ both in $S_d\backslash S_{1,d}$ such that

21 $$\begin{aligned} h:=2^{2^{k_1}}+m_1!-2^{2^{k_2}}-m_2! \ne 2^{2^{k_3}}+m_3!-2^{2^{k_4}}-m_4!=:h'. \end{aligned}$$

Let $\mathcal {P}$ be the set of possible prime factors of $d \in B$ which exceed $\log x$. We shall prove that $|\mathcal {P}| = \mathcal {O}((\log x)^5)$. For $h,h'$ in (21) we have that they are both divisible by *d* and thus $d|h-h'$. Every prime factor of *d* (in particular the ones larger than $\log x$) divideswhere the product is taken over all $m_i$ with $m_i!\le x$ and all $k_i$ with $2^{2^{k_i}}\le x$ for $i=1,2,3,4$. The dash indicates that the product is to be taken over the non-zero factors only. Since each factor in this product is of size $\mathcal {O}(x)$ any of these factors has at most $\mathcal {O}(\log x)$ prime factors. Furthermore, for the octuple $(k_1,k_2,k_3,k_4,m_1,m_2,m_3,m_4)$ we have  choices and altogether we have that $|\mathcal {P}|=\mathcal {O}((\log x)^5)$. Write $d=u_dv_d$, where $u_d$ is divisible by primes $p \le \log x$ only. Hence the factor $v_d$ is divisible only by primes in $\mathcal {P}$. Then

$$\begin{aligned} \sum _{d\in B} \frac{|(S_d\backslash S_{1,d})|}{d}\le \left( \sum _{\begin{array}{c} u \text { odd} \\ \mu (u)^2=1 \\ P(u)<\log x \end{array}} \frac{|(S_u\backslash S_{1,u})|}{u} \right) \left( \sum _{\begin{array}{c} v \text { odd} \\ \mu (v)^2=1 \\ p\mid v\Rightarrow p\in \mathcal {P} \end{array}} \frac{1}{v}\right) , \end{aligned}$$

where we used that $S_d\backslash S_{1,d}\subset S_u\backslash S_{1,u}$ if $u\mid d$. For the second sum we have

$$\begin{aligned} \sum _{\begin{array}{c} v \text { odd} \\ \mu (v)^2=1 \\ p\mid v\Rightarrow p\in \mathcal {P} \end{array}} \frac{1}{v}=\prod _{p \in \mathcal {P}}\left( 1+\frac{1}{p}\right) =\mathcal {O}(1), \end{aligned}$$

which follows from partial summation and the fact that $\mathcal {P}$ has $\mathcal {O}((\log x)^5)$ elements all larger than $\log x$. It thus remains to bound

$$\begin{aligned} \sum _{\begin{array}{c} u \text { odd} \\ \mu (u)^2=1 \\ P(u)<\log x \end{array}} \frac{|(S_u\backslash S_{1,u})|}{u}. \end{aligned}$$

For this, we fix $(m_1,m_2)$ with $m_1>m_2$ not both in $\{0,1\}$. Then putting $M_{1,2}=m_2!-m_1!$, we need to count the number of $(k_1,k_2)$ such that $2^{2^{k_1}}-2^{2^{k_2}}\equiv M_{1,2} \pmod u$. Analogously as before, for fixed $k_2$, this puts $k_1$ into a fixed arithmetic progression modulo *t*(*b*(*u*)). The number of $k_1$ with $2^{2^{k_1}}\le x$ in this progression is of order . Thus, we have

$$\begin{aligned}&\sum _{\begin{array}{c} u \text { odd} \\ \mu (u)^2=1 \\ P(u)<\log x \end{array}} \frac{|(S_u\backslash S_{1,u})|}{u} \ll \left( \frac{\log x}{\log \log x}\right) ^2(\log \log x) \\&\qquad \times \left( \log \log x \sum _{\begin{array}{c} u \text { odd} \\ \mu (u)^2=1 \\ P(u)<\log x \end{array}}\frac{1}{ut(b(u))}+\sum _{\begin{array}{c} u \text { odd} \\ \mu (u)^2=1 \\ P(u)<\log x \end{array}}\frac{1}{u}\right) \ll (\log x)^2. \end{aligned}$$

Here, we used Lemma 2 and Mertens’ formula, which yields

$$\begin{aligned} \sum _{\begin{array}{c} u \text { odd} \\ \mu (u)^2=1 \\ P(u)<\log x \end{array}}\frac{1}{u} = \prod _{3 \le p \le \log x} \left( 1+\frac{1}{p}\right) \ll \log \log x. \end{aligned}$$

$$\square $$

### Proof of Theorem 3

Since the density of integers of the form $p+2^{2^k}+m!$, $p \in \mathbb {P}$, $m,k \in \mathbb {N}$ and $m < 2^{2^6}-1$ is zero, we may suppose that $m \ge 2^{2^6}-1$. In this case, we have $m! \equiv 0 \bmod 2^{2^6}-1$, and for $k \ge 6$, we have that $2^{2^k} \equiv 1 \bmod 2^{2^6}-1$. If $n \equiv a+1 \bmod 2^{2^6}-1$, where *a* is a residue class $\bmod \, 2^{2^6}-1$ with $(a,2^{2^6}-1)>1$, then $(n-2^{2^k}-m!,2^{2^6}-1)>1$ which leaves only finitely many choices for the prime $p=n-2^{2^k}-m!$. This implies that the proportion of such *n* with a representation of the form $n=p+2^{2^k}+m!$ is zero. We have $2^{2^6}-1-\varphi (2^{2^6}-1)$ choices for the residue class *a* and half of the integers in these residue classes are odd which yields a density of

$$\begin{aligned} \frac{2^{2^6}-1-\varphi (2^{2^6}-1)}{2 \cdot ( 2^{2^6}-1)} = \frac{615850829669273873}{2459565876494606882}. \end{aligned}$$

We note that a more refined version of the above argument was used by Habsieger and Roblot [11, Sect. 3] to prove an upper bound on the proportion of odd integers not of the form $p+2^k$. $\square$

### Proof of Theorem 4

We will show that none of the integers *n* satisfying the following system of congruences is of the form $p+2^{2^k}+m!:$

$$\begin{aligned} 1&\bmod 2&1&\bmod 3&3&\bmod 5 \\ 2&\bmod 7&6&\bmod 11&3&\bmod 17 \\ 7&\bmod 19&9&\bmod 23. \end{aligned}$$

By the Chinese Remainder Theorem, the arithmetic progressions above intersect in a unique arithmetic progression. Let *n* be an element of this progression and suppose that $n=p+2^{2^k}+m!$.

If $m \ge 3$, then $n=p+2^{2^k}+m! \equiv p+2^{2^k} \bmod 3$. All primes except for 3 are in the residue classes $1,2 \bmod 3$ and $2^{2^k} \equiv 1 \bmod 3$ for $k \ge 1$. Thus, for $m \ge 3$ and $k \ge 1$ we have that $n = p+2^{2^k}+m! \equiv 1 \bmod 3$; hence, the only possible choice for *p* is $p=3$.

Next, we show that if $p=3$, then $m<5$. To do so, we use that $2^{2^k}\equiv 1 \bmod 5$ for $k\ge 2$; hence for $m \ge 5$ we are left with $n=3+2^{2^k}+m! \equiv \{0,2,4\} \bmod 5$, a contradiction to $n \equiv 3 \bmod 5$.

In the case that $k=0$, we will show that $m \ge 3$ implies $m<7$. Let $n=p+2+m!$ and $m \ge 3$. Then $n \equiv 1 \bmod 3$ implies that $p \equiv 2 \bmod 3$. If additionally $m \ge 7$, then $n=p+2+m! \equiv p+2 \bmod 7$. Since $n \equiv 2 \bmod 7$, the only possible choice for *p* is $p=7$, which contradicts $p \equiv 2 \bmod 3$.

Using the above observations, the only cases we need to consider are those of $m=0$, $m=1$, $m=2$, $m=3,4$ and $k=0$ or $p=3$ and $m=5,6$ and $k=0$.

If $m\in \{0,1\}$ and we additionally have that *p* is odd, then $n=p+2^{2^k}+1$ is even, a contradiction to $n \equiv 1 \bmod 2$. It remains to deal with the case when $p=2$. Then we have $n=2+2^{2^k}+1$ and we get a contradiction from $n \equiv 3 \bmod 5$ which would imply that $2^{2^k} \equiv 0 \bmod 5$.

For the case $m=2$, we use that $2^{2^k} \equiv 1 \bmod 17$ for $k\ge 3$. Hence, for $m=2$ and $k \ge 3$, we have that $n=p+2^{2^k}+2 \equiv p+3 \bmod 17$ which together with $n \equiv 3 \bmod 17$ leaves us with $p=17$. We use that $n=17+2^{2^k}+2 \equiv 2 \bmod 3$ to get a contradiction to $n \equiv 1 \bmod 3$. Since $m=2$ and $k=0$ imply $n=p+4 \equiv p+1 \bmod 3$, the only possible choice for *p* in this case is $p=3$ but $n=7 \not \equiv 3 \bmod 5$. If $m=2$ and $k=1,$ then $n=p+6$ and $n \equiv 6 \bmod 11$ implies that $p=11$. This contradicts $n \equiv 1 \bmod 3$. Last we need to deal with $m=2$ and $k=2$. In this case, $n=p+18 \equiv p+3 \bmod 5$, and hence, $n \equiv 3 \bmod 5$ implies that $p=5$. Now $n=23$ does not satisfy the congruence $n \equiv 1 \bmod 3$.

If $m=3$ and $p=3$ we have that $n=9+2^{2^k} \equiv {8,10,11,13} \bmod 17$ contradicting $n \equiv 3 \bmod 17$. On the other hand, if $m=3$ and $k=0$, then $n=p+8 \equiv p+3 \bmod 5$ and we get a contradiction as shown above.

For $m=4$ and $p=3$ we get $n=27+2^{2^k} \equiv \{9,11,12,14\} \bmod 17$, a contradiction to $n \equiv 3 \bmod 17$. If $m=4$ and $k=0$, it follows that $n=p+26 \equiv p+7 \bmod 19$ which implies $p=19$ and $n=45$. This contradicts $n \equiv 3 \bmod 5$.

In the case when $m=5$ and $k=0$, we have that $n=p+122 \equiv p+3 \bmod 17$. Together with $n \equiv 3 \bmod 17$ this only leaves $p=17$ which contradicts $n \equiv 3 \bmod 5$.

Finally, if $m=6$ and $k=0$, then $n=p+722 \equiv p+9 \bmod 23$. Together with $n \equiv 9 \bmod 23$, this only leaves $p=23$ which yields a contradiction to $n \equiv 3 \bmod 5$. $\square$

## Integers of the form $p+2^{2^k}+2^q$

### Lemma 5

The following estimate holds:

$$\begin{aligned} \sum _{n \le x}r_2(n) \gg x. \end{aligned}$$

### Proof

The lemma follows fromBy the Prime Number Theorem, we haveTogether withthis finishes the proof of the lemma. $\square$

### Lemma 6

The following estimate holds:

$$\begin{aligned} \sum _{n \le x}r_2(n)^2 \ll x. \end{aligned}$$

### Proof

Again $r_2(n)^2$ counts the number of solutions of the equation

$$\begin{aligned} p_1+2^{2^{k_1}}+2^{q_1}=p_2+2^{2^{k_2}}+2^{q_2} \end{aligned}$$

in $p_1,p_2,k_1,k_2,q_1$ and $q_2$ where $p_1+2^{2^{k_1}}+2^{q_1} \le x$. This means counting pairs of primes $(p_1,p_2)$ such that $p_2=p_1+h$, where

$$\begin{aligned} h:=2^{2^{k_1}}+2^{q_1}-2^{2^{k_2}}-2^{q_2}. \end{aligned}$$

If $h=0$ then either $(k_1,q_1)=(k_2,q_2)$ or w.l.o.g. $k_1>k_2$ and

$$\begin{aligned} 2^{2^{k_2}}\left( 2^{2^{k_1}-2^{k_2}}-1\right) =2^{q_1}\left( 2^{q_2-q_1}-1\right) . \end{aligned}$$

Since $2^{2^{k_1}-2^{k_2}}-1$ and $2^{q_2-q_1}-1$ are odd, we have that $2^{k_2} = q_1$ and hence $k_2=1$ and $q_1=2$. This leads to $2^{k_1} = q_2$ and hence to $k_1=1$ and $q_2=2$ a contradiction to $k_1>k_2$. If $h=0$ we thus have that $(k_1,q_1)=(k_2,q_2)$ and $p_2$ is fixed by a choice of $p_1,k_1$ and $q_1$. The last three parameters may be chosen in $\mathcal {O}(x)$ ways and we can deal with the contribution of solutions of the equation $p_2=p_1+h$ where $h \ne 0$. Since *h* is even, we may directly use the sieve bound from [15, Theorem 7.3] which, after summing over all *h*, yields an upper bound of order22for the sum in the lemma, where the dash indicates that $(k_1,q_1) \ne (k_2,q_2)$. Noting that the contribution of the prime 2 is just a constant factor, we disregard it. Furthermore $h \le x$ by definition, and a very crude upper bound for the number of prime factors of *h*, in particular for those larger than $\log x$, is given by . We thus get23If we fix $k_1,q_1$ and $k_2$, then the fact that $d\mid h$ implies

$$\begin{aligned} 2^{q_2} \equiv 2^{2^{k_1}}+2^{q_1}-2^{2^{k_2}}=:l \bmod d, \end{aligned}$$

where *l* is a fixed residue class $\bmod \ d$. This puts $q_2$ in a fixed residue class $\bmod \ t(d)$. Since we are counting representations of integers $n \le x$, we have . Hence if $t(d) > \log x$ there are at most two choices for $q_2$. If $t(d) \le \log x$, the Brun–Titchmarsh inequality yields an upper bound offor the number of choices of $q_2$. We thus get an upper bound of the following order for (23)24As earlier, by Mertens’ formula

$$\begin{aligned} \sum _{\begin{array}{c} d \text { odd} \\ P(d)\le \log x \end{array}}\frac{\mu (d)^2}{d} \ll \log \log x. \end{aligned}$$

To deal with the first sum in (24), we use  (see [17, Theorem 15]) and split the range of summation in two parts and getBy a result of Erdős and Turán [7, 8], the sums

$$\begin{aligned} \sum _{d \text { odd}} \frac{\log \log t(d)}{dt(d)} \text { and } \sum _{d \text { odd }}\frac{\log \log t(d)}{d\sqrt{t(d)}} \end{aligned}$$

converge which altogether proves an upper bound of order $\mathcal {O}((\log x)^2)$ for (23) and hence an upper bound of order $\mathcal {O}(x)$ for (22). $\square$

### Proof of Theorem 5

We prove the theorem by showing that the subset of positive integers in the residue class $3 \bmod 6$ having a representation of the form $p+2^{2^k}+2^q$ has density 0.

If $k>0$, then $2^{2^k}=4^{2^{k-1}}$. The fact that $4^2 \equiv 4 \bmod 6$ puts the term $2^{2^k}$ into the residue class $4 \bmod 6$ if $k>0$. Using the same fact again, we get for $q=2l+1$

$$\begin{aligned} 2^q =2^{2l+1}=2\cdot 4^l \equiv 2 \bmod 6. \end{aligned}$$

Furthermore, all primes except 2 and 3 are in the residue classes $\{1,5\} \bmod 6$. Thus if *n* is in none of the sets

$$\begin{aligned}&S_1:=\{p+2+2^q: p,q\in \mathbb {P}\}, \\&S_2:=\{p+2^{2^k}+4: p\in \mathbb {P}, k \in \mathbb {N}\},\\&S_3:=\{2+2^{2^k}+2^q: k \in \mathbb {N},q\in \mathbb {P}\},\\&S_4:=\{3+2^{2^k}+2^q: k \in \mathbb {N},q\in \mathbb {P}\}, \end{aligned}$$

all of which have density 0, and if *n* has a representation of the form $n=p+2^{2^k}+2^q$, then *n* is in one of the residue classes

$$\begin{aligned} \{1,5\}+\{4\}+\{2\} =\{1,5\} \bmod 6. \end{aligned}$$

The set

$$\begin{aligned} S=\{n \in \mathbb {N}: n \equiv 3 \bmod 6\} \backslash (S_1\cup S_2 \cup S_3 \cup S_4) \end{aligned}$$

has density , consists of odd integers only and none of its members is of the form $p+2^{2^k}+2^q$. This proves the first part of the Theorem.

To find a full arithmetic progression of integers not of the form $p+2^{2^k}+2^q$, we will add additional congruences ruling out the integers in the sets $S_1$, $S_2$, $S_3$ and $S_4$. We claim that none of the integers *n* satisfying the congruences

$$\begin{aligned} 3&\bmod 6&4&\bmod 5&4&\bmod 7 \\ 9&\bmod 13&5&\bmod 17&8&\bmod 19 \\ 20&\bmod 23&2&\bmod 29&3&\bmod 31 \\ 10&\bmod 37 \end{aligned}$$

is of the form $p+2^{2^k}+2^q$. By the above considerations, it suffices to check that none of the integers in the sets $S_1$, $S_2$, $S_3$ and $S_4$ is contained in this arithmetic progression.

We start with the integers in $S_1$. Take $n=p+2+2^q \in S_1$ and suppose that *n* is in the arithmetic progression constructed above. We use that except for $q\in \{2,3\}$, we have that $q \equiv \{1,5,7,11\} \bmod 12$ and that for any $l\in \mathbb {N}_0$ we have that

$$\begin{aligned} 2^{12l+1} \equiv 2^{12l+5} \equiv 2 \bmod 5 \text {, } 2^{12l+7} \equiv 2 \bmod 7 \text {, } 2^{12l+11} \equiv 7 \bmod 13. \end{aligned}$$

If $q \equiv \{1,5\} \bmod 12$, then $n=p+2+2^q \equiv p+4 \bmod 5$. Since $n \equiv 4 \bmod 5$, this implies that $p=5$. Now $7+2^{12l+1} \equiv 2 \bmod 7$ and $7+2^{12l+5} \equiv 0 \bmod 13$, contradiction to $n \equiv 4 \bmod 7$ and $n \equiv 9 \bmod 13$. In the case of $q=12l+7$, we get $n=p+2+2^{12l+7} \equiv p+ 4 \bmod 7$ and the only possible choice for *p* is $p=7$. Then $9+2^{12l+7} \equiv 2 \bmod 5$, a contradiction to $n \equiv 4 \bmod 5$. Finally if $q=12l+11$, then $n = p+2+2^{12l+11} \equiv p+9 \bmod 13$ and from $n \equiv 9 \bmod 13$ we get $p=13$. Since $n=15+2^{12l+11} \equiv 3 \bmod 5$, we again get a contradiction to $n \equiv 4 \bmod 5$. To finish off the integers in the set $S_1$, it remains to deal with $q \in \{2,3\}$. If $q=2$ we have $n=p+6 \equiv p \bmod 6$. Since $n \equiv 3 \bmod 6$, we are left with $p=3$ and $n=9$ which contradicts to $n \equiv 4 \bmod 7$. If $q=3$ then $n=p+10$ and from $n \equiv 10 \bmod 37$, we need to have that $p=37$, and hence $n=47$. This is impossible since it contradicts to $n \equiv 4 \bmod 5$.

Next, we deal with the integers in $S_2$ and we use that $2^{2^k} \equiv 1 \bmod 17$ for $k \ge 3$. Thus, for $k \ge 3$ and $n=p+2^{2^k}+4 \in S_2$ we have that $n=p+2^{2^k}+4 \equiv p + 5 \bmod 17$. From $n \equiv 5 \bmod 17$, we see that the only admissible choice for *p* is $p=17$, and hence, $n=21+2^{2^k}$. As above we use that $2^{2^k} \equiv \{2,4\} \bmod 6$ and thus $21+2^{2^k} \equiv \{1,5\} \bmod 6$ a contradiction to $n \equiv 3 \bmod 6$. We are left with $k \in \{0,1,2\}$. For $k=0$, we get $n=p+6$ which was ruled out when we dealt with the integers in $S_1$. If $k=1$ we have $n=p+8$ and from $n\equiv 8 \bmod 19$, the only possible choice for *p* is $p=19$ and thus $n=27$. This contradicts to $n \equiv 4 \bmod 5$. Finally, if $k=2$ we have $n=p+20$ and from $n \equiv 20 \bmod 23$ we again are left with a single possible choice for *p*, namely $p=23$. Now $n=43$, contradicting to $n \equiv 4 \bmod 5$.

For integers *n* in the set $S_3$, we have $n=2+2^{2^k}+2^q$. If $q=2$ we have $n \equiv 2^{2^k} \bmod 6$ and again using that $2^{2^k} \in \{2,4\} \bmod 6$, we get a contradiction to $n \equiv 3 \bmod 6$. If *q* is odd, then $2^q \equiv 2 \bmod 6$. If furthermore $k=0$, then $n = 4+2^q \equiv 0 \bmod 6$, and if $k=1$, we get $n=6+2^q \equiv 2 \bmod 6$. In both cases this yields a contradiction to $n \equiv 3 \bmod 6$. For $k \ge 2$ and *q* odd, we have that $2^{2^k} \equiv \{16,24,25\} \bmod 29$ and $2^{q} \equiv \{2,3,8,10,11,12,14,15,17,18,19,21,26,27\} \bmod 29$. For $k \ge 2$ and *q* odd, it is thus true that $2^{2^k} +2^q \not \equiv 0 \bmod 29$ and thus $n=2+2^{2^k}+2^q \equiv 2 \bmod 29$ yields a contradiction in this case.

Finally, for integers in the set $S_4$ we apply a similar argument as for integers in the set $S_3$. For any prime *q* we have that $2^{q} \equiv \{1,2,4,8,16\} \bmod 31$, and for all $k \in \mathbb {N}_0$ we get $2^{2^k}\equiv \{2,4,8,16\} \bmod 31$. Again $2^{2^k}+2^q \not \equiv 0 \bmod 31$ for any prime *q* and any non-negative integer *k*. Thus, $n=3+2^{2^k}+2^q \equiv 3 \bmod 31$ yields a contradiction. $\square$

## Footnotes

1 Note that $x_1$ and $x_2$ in the non-trivial solutions in Theorem 7 are never both powers of 2.
